# Core and accessory genome architecture in a group of *Pseudomonas aeruginosa* Mu-like phages

**DOI:** 10.1186/1471-2164-15-1146

**Published:** 2014-12-19

**Authors:** Adrián Cazares, Guillermo Mendoza-Hernández, Gabriel Guarneros

**Affiliations:** Departamento de Genética y Biología Molecular, Centro de Investigación y de Estudios Avanzados del Instituto Politécnico Nacional (CINVESTAV IPN), Mexico City, Mexico; Departamento de Bioquímica, Facultad de Medicina de la Universidad Nacional Autónoma de México, Mexico City, Mexico

**Keywords:** Phage, Comparative genomics, Pangenome, Mu-like phages, Phage adaptation, Horizontal gene transfer

## Abstract

**Background:**

Bacteriophages that infect the opportunistic pathogen *Pseudomonas aeruginosa* have been classified into several groups. One of them, which includes temperate phage particles with icosahedral heads and long flexible tails, bears genomes whose architecture and replication mechanism, but not their nucleotide sequences, are like those of coliphage Mu. By comparing the genomic sequences of this group of *P. aeruginosa* phages one could draw conclusions about their ontogeny and evolution.

**Results:**

Two newly isolated Mu-like phages of *P. aeruginosa* are described and their genomes sequenced and compared with those available in the public data banks. The genome sequences of the two phages are similar to each other and to those of a group of *P. aeruginosa* transposable phages. Comparing twelve of these genomes revealed a common genomic architecture in the group. Each phage genome had numerous genes with homologues in all the other genomes and a set of variable genes specific for each genome. The first group, which comprised most of the genes with assigned functions, was named “core genome”, and the second group, containing mostly short ORFs without assigned functions was called “accessory genome”. Like in other phage groups, variable genes are confined to specific regions in the genome.

**Conclusion:**

Based on the known and inferred functions for some of the variable genes of the phages analyzed here, they appear to confer selective advantages for the phage survival under particular host conditions. We speculate that phages have developed a mechanism for horizontally acquiring genes to incorporate them at specific loci in the genome that help phage adaptation to the selective pressures imposed by the host.

**Electronic supplementary material:**

The online version of this article (doi:10.1186/1471-2164-15-1146) contains supplementary material, which is available to authorized users.

## Background

Comparison of closely related bacterial genomes has been useful for the study of genome evolution over small time scales and identification of lateral gene transfer events and strain-specific genes [1]. Comparative analysis of different strains of *Pseudomonas aeruginosa* has shown that their genomes are mosaics consisting of a conserved component (core genome) interrupted by blocks of variable genes (accessory genome) located in limited chromosomal locations [2]. It has been proposed that *P. aeruginosa* shapes its accessory genome to favor survival in a wide range of ecological niches, which represents a major evolutionary force influencing genome composition [2].

Comparative studies of tailed bacteriophage genome sequences have shown a pervasive mosaic genetic architecture, presumably arising from extensive horizontal exchanges and non-homologous genetic recombination of ancestral sequences (see [3], for a review). Interestingly, the genomes of several bacteriophage groups consist of conserved and variable genes [4–7], like those of bacteria, suggesting that they emerged by a similar evolutionary mechanism. Genes that encode interacting proteins, such as virion structural genes, are usually arranged in continuous modules rarely interrupted by non-homologous recombination. There is evidence for exchange of large blocks of genes that produce fully functional phages that are different from the inferred parents [8, 9]. A type of accessory genes are the so-called "Morons" (units of more DNA), usually single coding regions flanked by a transcription promoter and terminator that are inserted between two adjacent genes in related phages [3, 7]. The nucleotide composition of morons is usually different from that of adjacent genes arguing about the recent acquisition of the elements from a different source. In some cases morons are lysogenic conversion genes expressed from the repressed prophage and apparently conferring a selective benefit on the prophage by benefiting the host.

Bacteriophage Mu, which infects *Escherichia coli*, is the prototype of various temperate phages found in other bacterial species. Phages D3112 and B3 of *Pseudomonas aeruginosa*, resemble Mu in that they lysogenize by integrating their genomes at almost random positions in the host chromosome, their DNA replicates by transposition and their viral particles contain heterogeneous segments of host DNA attached at both termini of the phage genomes [10–12]. The viral particles of D3112 and B3 have isometric icosahedral heads like Mu, but their long flexible tails differ from the contractile tail typical of Mu [10, 12]. Although these three phage genomes follow the module order lysis-lysogeny, transposition-replication and virion morphogenesis [10, 12, 13], their sequence diverges at the nucleotide level. Indeed, D3112 and B3 are not closely related phages as they are homologous for only 7.5 kb near their right termini [11] and B3 presents a notable genetic rearrangement in the left arm of its genome relative to those of D3112 and Mu [12].

Mu-like prophage sequences have been described in *Haemophilus, Neisseria* and *Deinococcus* [13]. Recently, many more *P. aeruginosa* Mu-like phage genomic sequences have been filed in the public databases, but we are unaware of efforts to compare these genomes and investigate the degree of diversity existing among them. In this work we sequenced the genomes of two locally isolated *P. aeruginosa* Mu-like phages whose sequences were similar to those of the D3112 group. Analysis of these and other annotated genomes revealed that they bear a common set of conserved genes representing most of the genome and a smaller group of short variable genes located in several specific loci. Following the *P. aeruginosa* terminology, the common set of genes is called “the core genome”, and the group of variable ORFs, which is different for each phage, is called “the accessory genome”; the sum of core and accessory genomes is “the pangenome”. We speculate that the accessory genes are acquired by horizontal transfer and that they increase the survival capacity of the phage by improving its adaptation to the particular conditions imposed by the ecology of its host.

## Results and discussion

### General features of the phages PaMx73 and H70

**Figure 1:**
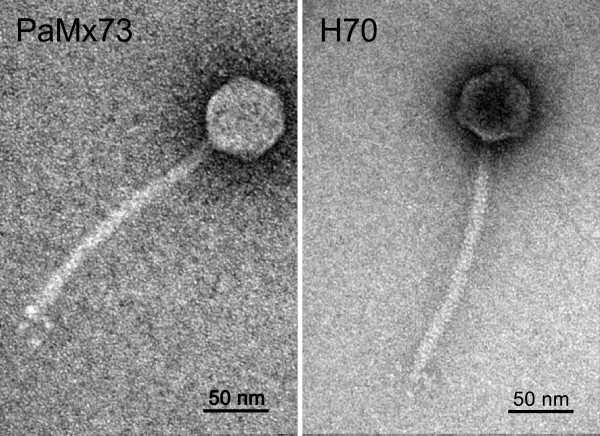
**Electron micrographs of PaMx73 and H70 phage particles.** The CsCl-purified phage particles were negatively stained with 2% uranyl acetate and visualized at 200,000-fold magnification.

**Figure 2:**
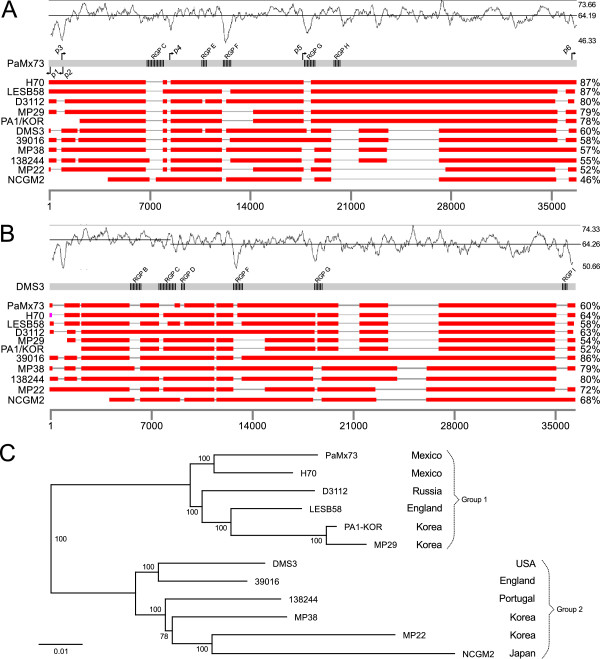
**Nucleotide level comparison of** ***P. aeruginosa*** **Mu-like phage genomes.** The *P. aeruginosa* Mu-like phage genomes of **A)** PaMx73 and **B)**, DMS3 were aligned with their relatives using BLASTn. The query genome is represented by the continuous grey bar and the subject genomes are displayed as discontinuous red bars. Location of Regions of Genomic Plasticity (RGPs) is indicated in each query genome. The fine grey line gaps in the red bars represent non homologous regions to the query genome. Some of these gaps coincide with lows in the graph of GC content distribution shown above the query genomes. The percentage of overall nucleotide sequence identity relative to the query genome is shown on the right margin for each phage sequence. The scale below the alignments represents length in nucleotides. Putative promoters identified in PaMx73 are marked with black arrows above and below the corresponding genome. **C)** Neighbor-joining tree of the *P. aeruginosa* Mu-like phage genomes based on a multiple alignment performed with the Mauve program. The numbers at the branch points indicate the bootstrap values represented as the percentage observed from 1000 replicas. The tree topology separates the compared phage genomes in two similarity groups whose prototypes are PaMx73 and DMS3. The country of isolation for each phage sequence is indicated on the right of figure along with the similarity group designation.

We studied two *Pseudomonas aeruginosa* temperate phages, PaMx73 isolated from an environmental water sample [14] and H70 rescued from a clinical strain. Both virions were morphologically siphophages with isometric heads (~54 nm in diameter) and long flexible tails (~163 nm in length) (Figure 1). Results of DNA sequence and gene organization of both phage genomes showed that they are homologous and largely syntenic, i.e., they share similar distribution of homologous genes (see below). The genome sequence of PaMx73 [14] had 56 ORFs in 36,570 bp and that of H70 presented 58 ORFs in 37,362 bp. The overall GC content of both genomes was 64%, slightly lower than the 66% of the *P. aeruginosa* host strain PA14 [15]. A combined search with different programs (see Methods) revealed six putative transcriptional promoters located at the same relative positions in each phage genome: p1 and p2 in the negative strand (transcribing leftward) and p3, p4, p5 and p6 in the positive strand (Figure 2A and Additional file 1). The promoters p1 and p6, located close to the genome ends are oriented outward suggesting that they would transcribe bacterial DNA next to the prophage ends. Not all the putative promoters showed typical -10 and -35 consensus sequences but most of them presented one or two binding motifs for *P. aeruginosa* transcriptional factors (Additional file 1).

### Sequence homology among PaMx73, H70 and Mu-like related genomes

BLASTn alignments showed that PaMx73 and H70 genomes were 87% identical to each other and homologous, to a lesser degree, with other *P. aeruginosa* phage genomes in public databases. These genomes corresponded either to vegetative phages (D3112 [10], MP29 [16], PA1/KOR/2010 [17], DMS3 [18], MP38 and MP22 [19]) or to putative prophages within bacterial genomes (LESB58 prophage 4 [20], 39016 [21], 138244 [22] and NCGM2 [23]) (Figure 2). All these genomes are organized in functional modules similar to those described for D3112, a temperate phage (Additional file 2) [10]. D3112 and MP22 are phages whose genome structures resemble that of coliphage Mu [10, 19]: a left module containing genes involved in the control of early gene expression and transposition-replication, a short middle regulatory region of late genes’ expression, and the right or late region containing morphogenesis genes. The putative promoter positions in the PaMx73 genome map (see above) also match most of the proposed promoter sites in Mu and D3112 genomes [13, 24]. Note that three of the analyzed genomes seemed incomplete as compared with the rest of the group (Figure 2 and Additional file 2): The putative prophage in NCGM2, which lacked around 2000 bp of the left end relative to the other genomes, probably due to a prophage partial deletion; PA1/KOR, about 2000 bp short in the left end [17]; and the genome of D3112 missing about100 bp at the right end, probably trimmed away during sequence assembly [10].

The mechanism of transposition-replication has been reported as the hallmark of the Mu-like phages [13, 25, 26]. In phage Mu, three imperfect repeat sequences close to both genome ends are recognized by its transposase [27, 28]. Therefore, we inspected the end regions of the phage genomes compared here for the presence of putative transposase binding sites. In all the genomes three 22 bp conserved putative binding sites were found in tandem next to the ends, except in the incomplete genomes mentioned above. The three binding sites at the left genome end, named L1, L2 and L3, corresponded to imperfect direct repeats located at 10, 93 and 124 bp from the end, whereas imperfect repeats R1, R2 and R3 were positioned at 4, 46 and 93 bp from the right genome end. In this case, R1 was inverted relative to R2 and R3 (Additional file 3). All the sequences and positions for the putative transposase binding sites were well conserved in consistency with the sequence conservation of the putative transposase A gene (see below).

The triplet 5'-TGT, identified at the genome ends of MP22 [19], has been reported as conserved in Mu-like phages of several bacterial groups [13, 25, 26]. We inspected the ends of the Mu-like phage genomes compared here looking for these terminal sequences. The triplet 5'-TGT was identified at the termini of PaMx73 and H70 during their genomic assemblies (see Methods) and, except for the incomplete genomes mentioned above, the triplet was present in all the genome ends examined in this work. The 5'-TG in the Mu genome has been shown to be important for assembly of a stable transposome complex [29, 30].

Short heterogeneous sequences, 50 to 100 nucleotides long, of *P. aeruginosa* genomic DNA were recognized flanking the genome ends of PaMx73 and H70 beyond the 5’-TGT sequences (data not shown). As in phage Mu, these host terminal sequences represent remnants of host DNA packaged in the virions, as relics of the mechanism of phage DNA replication by transposition [13]. Host DNA sequences as long as 2 kb have been reported at the right end of D3112 genome [10]. Thus, the shorter length of host DNA segments attached to the right end of the phage genomes was presumably an artifact resulting from the shotgun fragmentation process carried out on the phage DNAs before sequencing.

### The core and accessory genomes of the analyzed phages

All vs all BLASTn alignments of the twelve phage genomes indicated that long homologous segments were interspersed with short non-homologous regions, often located at the same relative positions (Figure 2A and B). The long homologous segments, mainly contained conserved ORFs over more than 90% identical among the compared genomes. In contrast, the non-homologous segments contained either different sets of short non-conserved ORFs or functionally conserved genes with heterogeneous sequences. The regularity of the similarity patterns, particularly clear in the genomic right arm regions of PaMx73 and DMS3 (Figure 2A and B), prompted us to organize the phage genomes in a neighbor joining tree (Figure 2C). The result showed two main branches, each with six phage genomes: group 1 represented by the genome of PaMx73 and group 2 by that of DMS3. The set of conserved genes will be referred to as the “core genome” and the short non-conserved ORFs will be denoted as the “accessory genome” (Additional file 2) following Mathee et al., for the genome structure described for *P. aeruginosa* [2]. The sites where the accessory genes were found will be called “Regions of Genomic Plasticity” or RPGs (Additional file 2) [2]. The sum of core and accessory genomes was named the “pangenome” of this group of phages [31]. As discussed below (Section Accessory genome), there seems to be more than a simple analogy between the concepts of phage and bacterial pangenomes.

Phage genomes containing core and variable components have been described for lambdoid phages [7], a group of T4-like phages [4] and cyanophages [5, 6]. The structure of the genomes described here formally parallels that of these phage genomes: the compared genomes share a core interrupted by several variable regions. In the T4-like group the core region primarily includes homologues of essential T4 genes, and the variable genome, located in specific loci named hyper plastic regions (HPRs), contains mostly small genes of unknown function. Nonetheless it is known that some of them encode adaptive functions that allow the phage to elude host exclusion systems (see below). It has been proposed that the core genes have evolved by vertical inheritance whereas the accessory genes have been horizontally transferred [4]. Thus, genomes with core and accessory components seem a common evolutionary strategy for both, temperate and virulent phages.

It has been speculated that variable gene regions in phage genomes are acquired by horizontal transfer and recombination at sites that do not interfere with the expression of essential genes [9]. Variable gene inserts often coincide with deviations in GC content along the genome indicating recent acquisition [10, 32, 33]. PaMx73 and DMS3 genomes showed an average GC content of 64.2% but two of their variable regions (see below, RGPs F and G) corresponded with valleys of GC content as low as 46.3% (Figure 2A and B). Inspection of the sequences flanking the different RPGs in the same genome, or the same type of RPGs in different genomes, did not lead to recognize sequences that could suggest a common recombinase target. As in the case of morons, the mechanism of acquisition of variable genes is mysterious [3].

### Assignment of functions to ORFs in the phage genomes

Based on homology to functional domains, and to amino acid sequences of phage proteins in the data bank, putative functions were assigned to fourteen ORFs in the genomes of PaMx73 and H70 (Additional file 2, bottom). These conserved ORFs were related to regulation of gene expression, DNA replication and virion morphogenesis.

**Figure 3:**
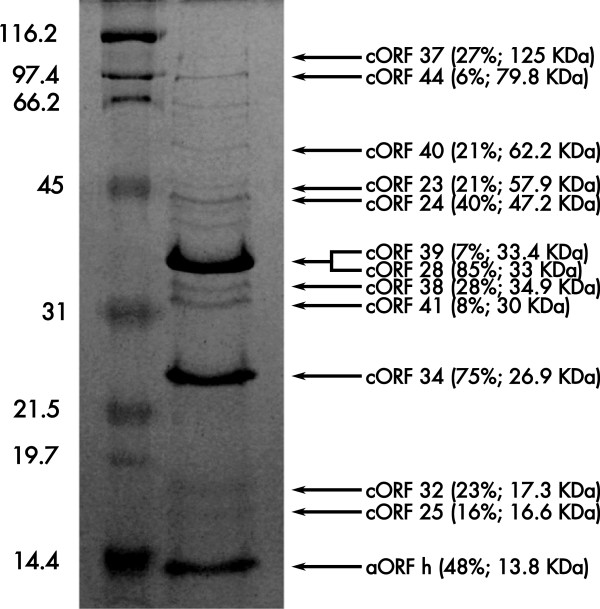
**Assignation of PaMx73 virion proteins by mass spectrometry analysis.** The protein components of PaMx73 purified virions were resolved by SDS-PAGE and stained with Coomassie brilliant blue. A molecular weight marker was included for reference in the left lane. The virion proteins were assigned by mass spectrometry to the corresponding ORFs (see Methods). All core ORFs are numbered as in Figure 4. ORF h was the only accessory ORF that was part of the virion. After the ORF numbers, in parenthesis, appears the percentage of amino acid sequence coverage obtained in the assay and the theoretical molecular weight of the identified protein.

**Figure 4:**
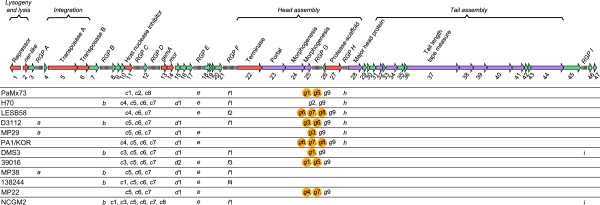
**Pangenome map of the phages examined.** The map represents the core genome of the phages analyzed here. The core genome is the collection of conserved ORFs represented by color arrows and numbered 1 to 47 below the map. Color code for arrows denotes: red, assigned function; green, unknown function, and purple, virion structural proteins identified by mass spectrometry in PaMx73 particles. The regions of genomic plasticity, RGPs A to I above the map (not to scale), indicate the location of the accessory genes in the pangenome. The individual accessory ORFs for each genome are indicated in low case font *a* to *i* below the corresponding RGPs. Anti-CRISPR-related genes [50, 51] detected by sequence homology are encircled in orange. Notice that accessory ORF *g*9 is associated with all known anti-CRISPR genes [50, 51]. Functions assigned by sequence homology to known phage proteins are indicated above the map. The functional modules in the pangenome are indicated by brackets on top of the figure.

To assign the structural proteins to specific ORFs in the PaMx73 genome, the proteins in the virion were analyzed by mass spectrometry. PaMx73 viral particles were banded in CsCl density gradients and their component proteins resolved through SDS-PAGE. The stained bands were eluted, trypsin digested and analyzed by tandem mass spectrometry (MS/MS) [34]. Thirteen proteins were identified as products of PaMx73 genes as they matched with the predicted products of the phage genome (Figure 3). These results confirmed the proteins predicted by sequence homology for five ORF products (portal, major head, tail length tape measure and two morphogenesis proteins). The other eight proteins corresponded to genes located in the tail genome region, except accessory ORF h located in the head module (Figure 4). To obtain further insights into the function of the newly identified virion structural proteins, we used the I-TASSER platform to predict their 3D structures and functions by homology to physically solved protein structures [35] (Additional file 4). Quality 3D models (see Methods) were obtained for the virion structural proteins encoded in ORF 32 (similar to a tail terminator protein), ORFs 38 and 39 (homologous to tail spike proteins), ORF 40 (match to an eukaryotic protein), ORF 44 (match to a bacterial glycoside hydrolase) and ORF *h* (similar to the head decoration protein, lambda gpD [36], see Section Accessory genome) (Additional file 4). ORFs 34 and 41 did not have significant 3D models. The tail termination protein of phage lambda stops polymerization of the tail tube when the precise tail length is reached and subsequently provides the surface of interaction with the virion head [37]. The tail spike proteins of several phages play key roles in the host cell recognition and DNA entry by binding and cleaving the primary receptors of the cell wall [38–40]. In addition, it has been reported that some phage tails carry glycoside hydrolase domains to degrade the cell wall previous to DNA entry [41].

### Core genome

The BLASTp comparative analysis of the twelve phage genomes examined here revealed that 47 ORFs are conserved in most of the phages (i.e. the core genome) (Figure 4, Additional files 2 and 5), except for the three incomplete genomes mentioned above (see section Sequence homology). Of these, 21 ORFs had assigned functions (Figure 4, red and violet arrows) whereas 26 ORFs remained unassigned (Figure 4, green arrows). A list of putative functions encoded by the core ORFs is presented in Additional file 6. Twelve of the thirteen proteins identified by mass spectrometry in the PaMx73 virion were assigned to specific ORFs in the core genome since they had homologs in all the Mu-like genomes analyzed (Figure 4, violet arrows). In contrast, the virion protein encoded by gene *h* (Figure 3), located between core ORFs 27 (protease-scaffold) and 28 (major head subunit protein) (RGP H, Figure 4), had homologs only in the genomes of similarity group 1 (Figure 2C). We suggest that gene *h* could have been lost in the genomes of similarity group 2 from a group 1 ancestor because it encodes a structural homolog of a head decoration protein (see above and section Accessory genome), therefore it seems to belong to the head module of the phage genome.

Most homologous core ORFs were 70 to 100% identical at the amino acid level among the compared genomes, but core ORFs 28, 29, 35, 36, 37, were highly similar only among members of each of the two similarity groups (Figure 2) and with lower sequence identity levels (43-60%) between members of different similarity groups. Additionally, there were ORFs that showed poor overall sequence conservation even among the members of the same similarity group. These ORFs, corresponding to the putative repressors, Ner-like proteins and terminases, exhibited 54%, 65% and 36% amino acid sequence identity, respectively. Other putative gene products with variable sequence, but unassigned function, were those encoded by core ORFs 4 and 45 (Figure 4). High variability of repressor and antirepressor (*ner*) sequences has been observed in the Stx-like coliphages [42] whereas the sequence variability between the repressors of D3112 and MP22 has been associated with the absence of cross-immunity [19]. Thus, the variation among the putative repressors observed here, likely indicates that these *P. aeruginosa* Mu-like phages belong to different immunity groups.

### ORFs previously overlooked in the phage genomes

**Figure 5:**
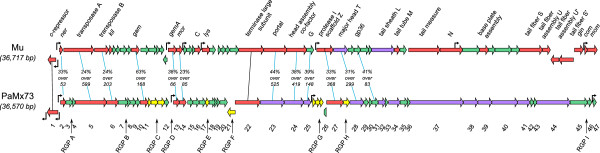
**Genome comparison between** ***Escherichia coli*** **phage Mu and PaMx73.** The genome maps of phage Mu, modified from [13], and PaMx73 are drawn to scale. The ORFs in both maps are represented by color coded arrows, as in Figure 4, adding the yellow arrows that represent accessory ORFs. Putative promoters identified in PaMx73 and those reported for Mu [13] are marked with angled black arrows. Some genes above phage Mu map are labeled with the functions that they encode [13]. Core ORFs in PaMx73 genome are numbered and the position of the regions of genome plasticity, RGPs A to I is indicated. The ORFs of Mu and PaMx73 were compared using BLASTp and the ORFs showing sequence similarity are connected by cyan lines between the two maps. The percentages of sequence similarity between the connected genes are indicated. Black lines connect ORFs encoding the same function, but showed no sequence homology.

The comparative analysis of a group of homologous genomes facilitates detection of errors and improves annotations [1]. The gene-by-gene comparison results and the synteny shared by the Mu-like phage genomes, allowed us to identify 46 ORFs that had been overlooked in the original annotations of the ten genomes obtained from the data bases (Additional file 7). These previously unidentified ORFs encoded either core or accessory genes (Additional file 2). In other cases it was noticed that a reported ORF might be shorter than homologous ORFs due to stop codons likely created by sequencing or assembly mistakes (Additional file 7). Additionally, based on synteny and similarity with genes of phage Mu and the presence of functional domains, we assigned the functions of GemA, Mor and terminase to the core ORFs 13, 14 and 22, respectively (Figure 5 and Additional file 6). Note that these functional assignations represent revised versions of the original annotations in other phage genomes of this group [10, 19].

### Accessory genome

The accessory genome of the revised Mu-like phages contained mainly ORFs ranging from 34 to 100 codons that were either phage specific or shared by several phage genomes (Figure 4 and Additional file 6). The accessory ORFs were always located in the phage genome at positions that corresponded to non-homologous regions in the nucleotide sequence alignments (see Figure 2A and B). These regions of genomic plasticity (RPGs) were mainly located in the left arm of the genomes and each contained from zero to six ORFs (Figure 4 and Additional file 2). Nine different RPGs were labeled ‘A to I’ in the genome maps and the different accessory ORFs recognized within each RGP were identified by the corresponding lower case letter and consecutive number (Figure 4). Each genome contained between 7 and 11 accessory ORFs distributed among 4 to 7 RGPs. Note that with the exception of RGPs C and G, the remaining regions contain only one ORF and, therefore, they could be considered as regions of insertion-deletion or “indels”. These accessory genes could be examples of the morons described in lambdoid phages [3, 7]. One interesting example of morons increasing the phage fitness by aiding virion stability is provided by the genes encoding capsid decoration proteins in different phages [3]. These genes have been considered accessory elements since they are absent in very closely related genomes and may be advantageous for the virions under certain conditions [3, 43]. For example, gpD and Dec proteins in lambdoid phages confer virion stability against chelating agents [43, 44], gpD provides mechanical reinforcement to withstand external physical stress in lambda [45] and Soc in T4 confers capsid resistance against high pH and thermal challenges [46, 47]. Despite proteins h of PaMx73 and gpD of lambda do not show sequence similarity, their genes are syntenic, ie., they are located between the genes encoding protease-scaffold and major head proteins in the corresponding genomes [13, 44]. In addition, proteins h, gpD and the decoration protein Dec of phage L, also present similar molecular mass (~11-14 KDa, Figure 3; [43, 44]). Based on the above coincidences, we suggest that protein h represents a new capsid decoration protein although additional experimental characterization will be necessary to validate this proposal.

Overall, 28 types of accessory ORFs were identified (Figure 4). The estimated size of the accessory genome in each phage represented between 6 to 10% of the genome. These non-homologous regions may have been acquired by recombination mediated by Red-like functions. Short sequences with as little as 78% of identity are used by Red-like functions to recombine short DNA segments into lambdoid phage genomes [48]. A detailed analysis of the sequences flanking the accessory ORFs in Mu-like genomes is needed to speculate about the mechanism of heterologous gene gaining. Furthermore, it has been shown that lambdoid phages encoding their own recombination system bear more mosaic genomes and possess more diverse gene repertoires than those lambdoid phages that do not encode any recombinase, thus increasing the phage diversity and facilitating the possible adaptation to the host [49].

Taking into account the number and type of accessory ORFs present in each genome, no two genomes were identical. The accessory ORFs *c*6 and *c*7 were shared by most phages, in contrast, ORFs *c*2, *d*2, *f*2, *f*3, *f*4, *g*2 and *g*5 were unique for a phage (Figure 4). It is possible that accessory genes in the Mu-like genomes are acquired by horizontal gene transfer and selected through the specific developmental history of each phage [9]. The majority of accessory ORFs recognized here seems to be restricted to *P. aeruginosa* Mu-like phage genomes (Additional file 6, Figure 6) and do not have an assigned function. However, some of them seem to confer selective advantages to the phage. It has been recently reported that genes 29 and 30 of phage D3112, corresponding to *g*3 and *g*4 in RGP G (Additional file 6), and gene 29 of phage MP29, corresponding to *g*3, encode proteins that inhibit the CRISPR/Cas system of *P. aeruginosa* [50, 51]. The presence of these accessory genes confers phage the advantage of infecting strains of *P. aeruginosa* harboring an active CRISPR-Cas system, a bacterial immunity system against phage infection [50, 51]. This function seems an adaptation to host conditions in an analogous way to the accessory genes encoding the internal proteins in phages P1 and T4 which prevent cell modification-restriction systems to act on the phage DNA targets [4, 52, 53]. Other putative anti-CRISPR proteins were encoded in *g*1, *g*5, *g*6, *g*7 and *g*8 in the phage genomes analyzed here [50, 51] (Figure 4, Additional file 6). Interestingly, RGP G coincided with regions of low GC content (Figure 2) implying that anti-CRISPR genes were acquired recently by horizontal transfer [10, 32, 33]. In addition, RGP F also coincided with regions of low GC content for phage genomes bearing *f*1, *f*3 and *f*4 genes.

**Figure 6:**
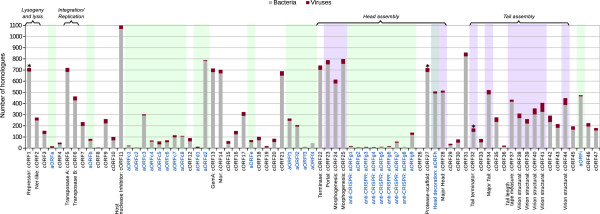
**Homologue frequencies for the Mu-like pangenome ORFs in the data bank.** The ORFs in the pangenome of the *P. aeruginosa* Mu-like phages were analyzed by BLASTp to determine the number of homologues in the non-redundant NCBI database. The number of homologues identified for each ORF in viruses (red bars) or bacteria (grey bars) is plotted on the graph. The core ORFs (cORF), corresponding to those in PaMx73 genome, are labeled in black whereas the accessory ORFs (aORF) of different phage genomes (see Additional file 6) are labeled in blue and the corresponding bars shaded in green. The bars corresponding to ORFs encoding virion proteins identified by mass spectrometry in PaMx73 are shaded in purple. Three ORFs matching eukaryota homologues are indicated with asterisks (*) above the corresponding bars. No homologues were found in Archaea. Functions inferred for some ORFs are under the corresponding label. Functional modules are indicated by brackets above the histogram and the gene order in the graph is represented as in the phage genomes.

RGP C, which clusters several ORFs, is reminiscent of the *nin*R region of phages λ, HK97, HK022 and P22 [7]. These phage genomes bear a group of about ten ORFs between genes P and Q. Like the ORFs in RGPs C, the genes in the *nin* region are short, less than 100 codons long, closely packed together, dispensable, and unique or shared only among some members of the group. As proposed for *nin* genes [7], function of genes in RGPs C and G may help phages to adapt to the particular host they infect. The genes *rex*A, *rex*B and *ren* of phage lambda may also represent examples of accessory genes conferring a selective advantage. The *rex* genes encode a two-component exclusion system that inhibits the growth of other phages infecting lambda lysogens [54]. The gene product of *ren* prevents lambda from self-exclusion [54]. It has been proposed that acquisition of novel metabolic capabilities in *P. aeruginosa* through horizontal gene transfer appears to be a key evolutionary force shaping the bacterial genome which is reflected in the genome plasticity of individual strains [2]. We and other authors [3] propose that a similar mechanism rules the genomes of the phages in their adaptation to the particular host exclusion functions.

### Distribution of homologues for the Mu-like pangenome ORFs in the data bank

To investigate about the nature and origin of core and accessory genes we looked for the frequency of homologous genes for each ORF through BLASTp searches against the non-redundant NCBI database (Additional file 6 and Figure 6). Bacterial and viral sequences accounted for almost all the homologues detected in the database (16559 and 3524, respectively). Several ORFs had homologues across a variety of bacterial species but, interestingly, the core genes had matches mainly from *Pseudomonas* genomes whereas the accessory genes had matches from the *Pseudomonas* genera and other most distant bacterial species. On average, the ORFs in the core genome had about three fold more homologues than the accessory ORFs (Figure 6 and Additional file 6) underscoring the essentiality of core gene functions. On the contrary, accessory ORFs generally had a lower number of homologues (Figure 6) with the exception of ORFs d2, h and i which showed about 500 matches or more each. Notice that these exceptions suggest that such ORFs could have been originally core genes that were lost from the phage genomes under conditions where they were dispensable. These ORFs represent interesting candidates to elucidate their function. ORF h, which may encode the capsid decoration protein in the Mu-like phages of the similarity group 1 (Figures 2 and 4), could represent a core head gene that was lost from the phage genomes of the similarity group 2 (Figure 4). Our analysis confirms the observation concerning the low number of homologues to anti-CRIPSR genes in the databases suggesting that they are specific for Mu-like phages and other mobile genetic elements of *P. aeruginosa* [50]. Note that these results are restricted to the sequences available in the non-redundant NCBI database, therefore a sampling bias exist for the homologues to core and accessory ORFs.

### Genome comparison between *P. aeruginosa*Mu-like phage PaMx73 and coliphage Mu

In spite the genomic homology observed among the *P. aeruginosa* Mu-like phages analyzed here, a BLASTn comparison between the genomic sequences of PaMx73 and coliphage Mu [13] revealed no significant sequence similarity. Yet, the two genomes showed similar functional modular organization. Based on BLASTp searches, twelve ORFs in the left arm of PaMx73 genome corresponded to homologous genes in the left arm of the Mu genome. These genes encode transcriptional regulation, replicative transposition and head morphogenesis proteins in Mu and show between 23 to 63% amino acid similarity with their corresponding homologues in PaMx73 (Figure 5). However, there were about twenty short ORFs without assigned functions in the Mu and PaMx73 left arm regions that did not show homology. Total genome comparison revealed that the right genomic arms of both phages were different in sequence, ORF number and size. This was expected because the right genome arms encode the tail genes and the two phages differ strikingly in tail morphology. Mu shows the contractile tail of myophages whereas PaMx73 has the flexible tail typical of siphophages (Figure 1). Interestingly, the genomic right arms of Mu and Mu-like myophages that infect other bacterial genera are homologous in gene distribution, size and sequence [13]. Since H70 (Figure 1), D3112 and MP22 phages have also been characterized as siphophages [10, 19], the flexible tail is apparently a unique feature of this group of *P. aeruginosa* Mu-like viruses. This appears to represent an interesting case of mosaicism between phages of different lineages.

We looked for three other genomic features of Mu in *P. aeruginosa* Mu-like phages: 1) The invertible G segment, 2) the in-frame translational start of the scaffolding protein within the protease gene and 3) the translational frameshift in the overlapping tail assembly genes [13]. As expected, no G invertible segment was identified among the Mu-like tail genes as the tail structure of this group of syphophages is totally different from the Mu tail. However, a putative internal start site in the protease-encoding gene 27 of PaMx73 was identified at Val 177 codon that could be the initiator of the scaffolding protein because it is preceded by a plausible Shine-Dalgarno sequence (ACGAGGA) by a 9 nucleotide spacer. In spite of the sequence divergence observed between these protease/scaffolding genes and those of Mu (33% of sequence similarity at the amino acid level), they show similar lengths, their internal start sites are located at the same relative positions and their corresponding Shine-Dalgarno sequences are placed to eight bases from the start codons (data not shown; [13]). Concerning the third feature, the putative overlapping tail assembly genes in PaMx73 could correspond to core ORFs 35 and 36. A slippery sequence T TTT TTC [50] was located at codons 116 to 118 of core ORF 35, 43 codons ahead of the stop codon (TAA) overlapping the core ORF 36 putative initiation codon (AUG). This configuration would require a -1 frameshifting to read both core ORFs as a unique gene conforming the majority of frameshift sequences analyzed for the tail assembly genes of many phages leaving the -2 frameshift for the Mu genes as exceptional [55].

## Conclusions

The genomic characterization of two locally isolated *Pseudomonas aeruginosa* bacteriophages showed that they belong to a family of phages and putative prophages of clinical strains reported worldwide, the prototype of which is D3112. The genomic nucleotide sequences of a dozen phages of this group were 50 to 90% identical among themselves, but in regard to the distribution of predicted protein sequences, they were highly syntenic. From a broader perspective, the genomic features indicated that the phages resembled coliphage Mu, however, their tail is flexible and not contractile like those of Mu and Mu-like phages of other bacterial species. The genomes compared here had long homologous regions interspersed with short heterologous blocks. The long conserved regions, which represent most of each genome, contained essential genes encoding replication and regulatory functions and structural proteins of the viral particles, whereas the genes located in the heterologous blocks were variable for each phage and presumably non-essential on the used plating host. This group of accessory genes, which seem to be acquired by horizontal transfer, may represent a selective advantage for the phages. Remarkably, among these were anti-CRISPR genes, which permit certain infections of hosts harbouring the CRISPR-Cas immunity system and a gene encoding a putative decoration protein that could be involved in the capsid stability. These observations extend to another group of phages the concept of “pangenome”, the sum of core and accessory genomes, not only in the way that they are distributed in the chromosomes, but also in their functional and evolutionary implications for phage biology.

## Methods

### Bacterial strains and bacteriophage isolation

The *Pseudomonas aeruginosa* clinical strains HIM5 and Ps33 were cultivated overnight with shaking at 37°C in Luria-Bertani (LB) medium. Bacteriophage PaMx73 was isolated from an environmental water sample [14] and bacteriophage H70 from the supernatant of a culture of the lysogenic strain HIM5 (Sepúlveda-Robles and Uc-Mass, personal communication). Strain Ps33 was the host to propagate PaMx73 and H70.

### Bacteriophage propagation, purification and electron microscopy

Bacteriophage propagation was performed using the standard soft agar overlay method [56]: 100 μl of phage stock (~10^8^ pfu) were mixed with 300 μl of *P. aeruginosa* liquid culture and 3 ml of LB top agar. The mixture was overlaid on a plate containing LB solid medium and then incubated overnight at 37°C to produce the confluent lysis of the host cells. The phage particles were recovered by scraping off the top agar layer and adding 5 ml of modified phage buffer (50 mM TrisHCL-pH 8, 10 mM MgSO4, 100 mM NaCl, and 0.01% Gelatine) to the surface of the plate. The agar-containing suspension was taken off the plate, stirred slowly during five hours at 4°C, and then centrifuged at 9300 g for ten minutes. The supernatant was treated with DNase I and RNase (1 μg/ml each, at 37°C for 30 min), and the phage particles were precipitated in 1.4 M NaCl and 16% w/v PEG 8000 at 4°C. The precipitated phage particles were concentrated by centrifugation at 8000 g for 30 min and subsequently purified by CsCl gradient centrifugation as previously described [56]. Dialyzed CsCl-purified phage stocks were used for electron microscopy. 10 μl of phage particles were deposited on a carbon-coated copper grid and incubated 5 min at room temperature. The excess solution was adsorbed with filter paper and the grid was stained twice with uranyl acetate (2%, pH 7) for 30 sec and 2 min, respectively. Grids were examined under a JEM-2000 transmission electron microscope at 80 Kv. Dimensions of the virions were calculated from 15 viral particles.

### Bacteriophage DNA extraction, sequencing and assembly

DNA was obtained by phenol-chloroform extraction from CsCl-purified phage suspensions as previously described [56]. High-throughput DNA sequencing was carried out at the National Laboratory of Genomics for Biodiversity (CINVESTAV, Irapuato, Mexico) using the Roche/454 system for PaMx73 [14], and the SOLiD technology for H70 DNAs. The 454 sequence reads were preprocessed with the Newbler assembler using default values (http://www.roche-applied-science.com) whereas SOLiD reads were preprocessed using the Applied Biosystems de novo assembly accessories. The phage genomes were assembled de novo using Velvet v1.1 [57], and refinement of the assemblies was performed by inspection. The reads mapping at the genome ends were trimmed from the final sequence until the last conserved nucleotide in all the cases.

### Genome annotation and sequence analysis

The coding sequences or in PaMx73 and H70 genomes were predicted with heuristic Hidden Markov Models using GeneMark v1.1 [58]. The location of ORFs positions was further corrected identifying ribosome binding sites with rbs_finder.pl [59]. Determination and visualization of GC contents were performed with Artemis [60]. BLASTp searches [61] against the non-redundant protein database on the NCBI server were carried out with the predicted ORF products to identify homologous sequences and improve the genome annotation. Conserved protein domains and protein families were searched with InterProScan [62] and NCBI-CDD [63]. The Artemis annotation tool [60] was used to conduct the functional genome annotation integrating BLAST, InterPro and CDD data. The non-coding regions of the phage genomes were screened for the presence of putative promoter sequences using BPROM (Softberry, Inc.) and Neural Network Promoter Prediction (NNPP) [64] programs. The promoter analysis tool hosted in PRODORIC website [65] was then used to scan the putative promoter sequences searching for transcription factor binding sites specific for *P. aeruginosa*.

### Accession numbers

The nucleotide genome sequences and annotations of PaMx73 and H70 were deposited in GenBank under accession numbers JQ067085 and KM233689, respectively.

### SDS-PAGE and mass spectrometry analysis of the virión structural proteins

CsCl-purified phage particles were resuspended in Laemmli loading buffer and boiled for 5 min. The mixture was loaded onto a 10% SDS-PAGE gel and the component proteins were resolved at 180 volt for 1.5 h. Protein bands were visualized by staining with Coomassie Brilliant Blue R250 dye and a pre-stained SDS-PAGE broad range protein standard (BioRad Hercules, CA, USA) was used to estimate the molecular weight of the observed proteins. The protein bands were carefully excised from the Coomassie-stained SDS gel and destained for 12 h with a mixture of 50% methanol and 5% acetic acid. The destained slices were washed with deionized water, soaked for 10 min in 100 mM ammonium bicarbonate, dehydrated with 100% acetonitrile and vacuum-dried. Proteins were reduced with 10 mM DTT and S-alkylated cisteine with 100 mM iodoacetamide in 100 mM ammonium bicarbonate. In-gel digestion was performed by adding 600 ng of mass spectrometry-grade trypsin (Promega, Madison, WI, USA) in 50 mM ammonium bicarbonate followed by overnight incubation at room temperature. Peptides were extracted twice with 50% acetonitrile and 5% formic acid for 30 min and the extracts were vacuum-dried and resuspended in 20 μL of 0.1% formic acid. Analysis of tryptic peptides was carried out using an integrated nano-LC_ESI_MS/MS system. Spectra were acquired in automated mode using data-dependent acquisition (DDA) and DDA raw data files were processed and subsequently converted to peak lists (pkl format) using the ProteinLynx Global Server v2.4 (PLGS) software (Waters Corporation). The mass spectra data in pkl files were compared with the putative protein sequences of PaMx73 using PLGS, OMSSA [66] and MASCOT (Version 1.6b9, Matrix Science, London; available at http://www.matrixscience.com) search algorithms to achieve the protein identification.

### Computational modeling of PaMx73 virion proteins

Virion structural proteins identified by mass spectrometry analysis but without function inferred by sequence homology were selected to predict their 3D structures and functions using the I-TASSER platform (http://zhanglab.ccmb.med.umich.edu/I-TASSER/). The putative amino acid sequences of the selected proteins were submitted for computational modeling to the I-TASSER web server following the procedure described [35]. 3D models with a minimal C-score of -3 or higher were considered reliable structures. Minimum TM-score and coverage values of 0.5 and 0.6, respectively, and functional congruence among the structural matches observed for each predicted model, were the criteria taken into account to consider a structural alignment as significant.

### Comparative genome analysis

Genomes with sequence homology to PaMx73 and H70 were found via BLASTn searches [61] against the nucleotide collection of NCBI. The homologous genomes were acquired from NCBI under accession numbers [GenBank:FM209186] (LESB58), [GenBank:NC_005178] (D3112), [GenBank:NC_011613] (MP29), [GenBank:HM624080] (PA1/KOR), [GenBank:NC_008717] (DMS3), [GenBank:CM001020] (39016), [GenBank:NC_011611] (MP38), [GenBank:AEVV01000017] (138244), [GenBank:NC_009818] (MP22) and [GenBank:AP012280] (NCGM2). Full lengths of putative prophage sequences (see the text, section Sequence homology) were determined by identifying the triplet 5'-TGT, conserved at termini of vegetative Mu-like phages, or detecting the last prophage ORF matching with the rest of compared genomes. Genomic comparisons at nucleotide level were performed with BLASTn to identify the extension and location of homologous regions. Percentages of nucleotide identity were calculated from alignments performed with MUMer v3.0 [67] and genome maps were constructed using in-house scripts. A neighbor joining tree was constructed based on a multiple genome alignment made with Mauve [68], using a progressive alignment with default settings. Homology searches at protein level were carried out following an all-versus-all strategy with BLASTp to identify the ORFs corresponding to core and accessory components of the phage genomes. Phage ORFs were considered homologous if they were syntenic among compared genomes and their BLASTp matches had a maximun e-value of 1e-05. Additionally, BLASTp searches were used to detect ORFs that were overlooked in the annotations of genomes acquired from NCBI. The previously overlooked ORFs were then considered to determine core and accessory genomes.

The number of homologues deposited in GenBank for each ORF in the pangenome was determined by BLASTp searches. The core ORFs of PaMx73 were used as query sequences for the core genome whereas the accessory ORFs of the different phage genomes were used as query to examine the accessory genome (Additional file 6). The similar sequences detected through the BLASTp searches were considered reliable homologues if the sequences shared at least 75% of their total length, with minimal similarity coverage of 75% of the total alignment and if the hit had a maximun e-value of 1e-03. The information about the organism harboring each homologue was used to classify them into the main categories: Viruses or Bacteria. Matches to vectors sequences were eliminated by inspection during the search process.

## Electronic supplementary material

Additional file 1: **Putative promoters found in PaMx73 and H70 genomes.** (PDF 47 KB)

Additional file 2: **Comparison of annotated** ***P. aeruginosa*** **Mu-like phage genomes.** (PNG 867 KB)

Additional file 3: **Transposase binding sites found in the phage genomes analyzed.** (PDF 80 KB)

Additional file 4: **Prediction of PaMx73 virion protein functions by computational modeling.** (PDF 19 KB)

Additional file 5: **Equivalence of core ORFs in the compared genomes.** (PDF 90 KB)

Additional file 6: **BLASTp searches of the ORFs encoded in the pangenome of the** ***P. aeruginosa*** **Mu-like phages analyzed.** (PDF 127 KB)

Additional file 7: **Overlooked ORFs in the GenBank files of** ***P. aeruginosa*** **Mu-like phages.** (PDF 95 KB)
