# Supplementary material for: Core and accessory genome architecture in a group of Pseudomonas aeruginosa Mu-like phages
Source: BMC Genomics. 2014 Dec 19;15(1):1146. doi: 10.1186/1471-2164-15-1146 (PMC4378225; doi:10.1186/1471-2164-15-1146)
Supplement: Supplementary file 1 — Additional file 1: Putative promoters found in PaMx73 and H70 genomes. (PDF 47 KB) [file 12864_2014_6884_MOESM1_ESM.pdf]

Additional file 1: Putative promoters found in PaMx73 and H70 genomes

Promoter	Position	Sequence	Scores		
			NNPP	BPROM	PRODORIC
**PaMx73**					
p1	148-99	cgatcaatattttgcagtccttgctcaatcctgatcgatt**T**tttccaaac	0.98		AlgU -10 (5.9)
p2	1008-959	gtgtctaacgtgacttagggcataaacatagcgagaaaat**C**ctcgcacag	0.94		PvdS (6.07), RhlR (8.83)
p3	951-1000	atccg**ttgctg**tgcgaggattttct**cgctatgtt**tatgcc**C**taagtcacg	0.99	48/47	
p4	8412-8461	ttcttcttcaccaacccggacggaacactcaaaaattcctg**T**aaagcctg	0.69		NarL (5.02)
p5	17626-17675	cggcaca**ttgtgc**ctattgcgtatt**aggcacaat**gtgcct**A**atctagcct	0.56	47/8	NarL (5.33)
p6	36188-36237	gataattgtgaccaacgtcgcctttttgctacggtcccca**G**ctgatgtgc	0.89		FleQ (7.15), RhlR (9.62)
**H70**					
p1	148-99	cgatcaatatctttcattccgcgcccaatcctgatcgtta**T**tttccaaac	0.98		Fur (9.07), AlgU -10 (5.9)
p2	1220-1171	gtgtctaacgtgacttagggcataaacatagcgagaaaat**C**ctcgcacag	0.94		PvdS (6.07), RhlR (8.83)
p3	1163-1212	atccg**ttgctg**tgcgaggattttct**cgctatgtt**tatgcc**C**taagtcacg	0.99	48/47	
p4	9525-9574	g**ttgccg**aaattgctgagcgactcg**cgtcacgtt**ttgccg**A**attgatcca		11/55	
p5	18721-18770	gtgccta**ttgatg**agattgcggatt**acgcacaat**gtgcct**A**atctagcct	0.70	33/52	NarL (5.33)
p6	36974-37023	ggtagttgtgatggcgttctcgtttttgccacgttccgaa**A**gtctgatgt	0.64		AlgU -10 (6.22)

Putative promoter sequences in the genomes of PaMx73 and H70 were searched using NNPP and Softberry BPROM programs. The promoters identified by at least one of the programs were subsequently scanned for the presence of *P. aeruginosa* transcriptional binding motifs using the database of Prodoric. The putative transcription starts are indicated in bold capitals. Sequences -10 and -35 are indicated in bold low case font only for those promoters identified by Softberry BPROM. The transcriptional binding motifs, identified with PRODORIC, are color coded in the promoter sequence and the name of their corresponding transcriptional activator. The scores obtained with each program are indicated in the respective columns.
