# Supplementary figures and images for: Core and accessory genome architecture in a group of Pseudomonas aeruginosa Mu-like phages
Source: BMC Genomics. 2014 Dec 19;15(1):1146. doi: 10.1186/1471-2164-15-1146 (PMC4378225; doi:10.1186/1471-2164-15-1146)

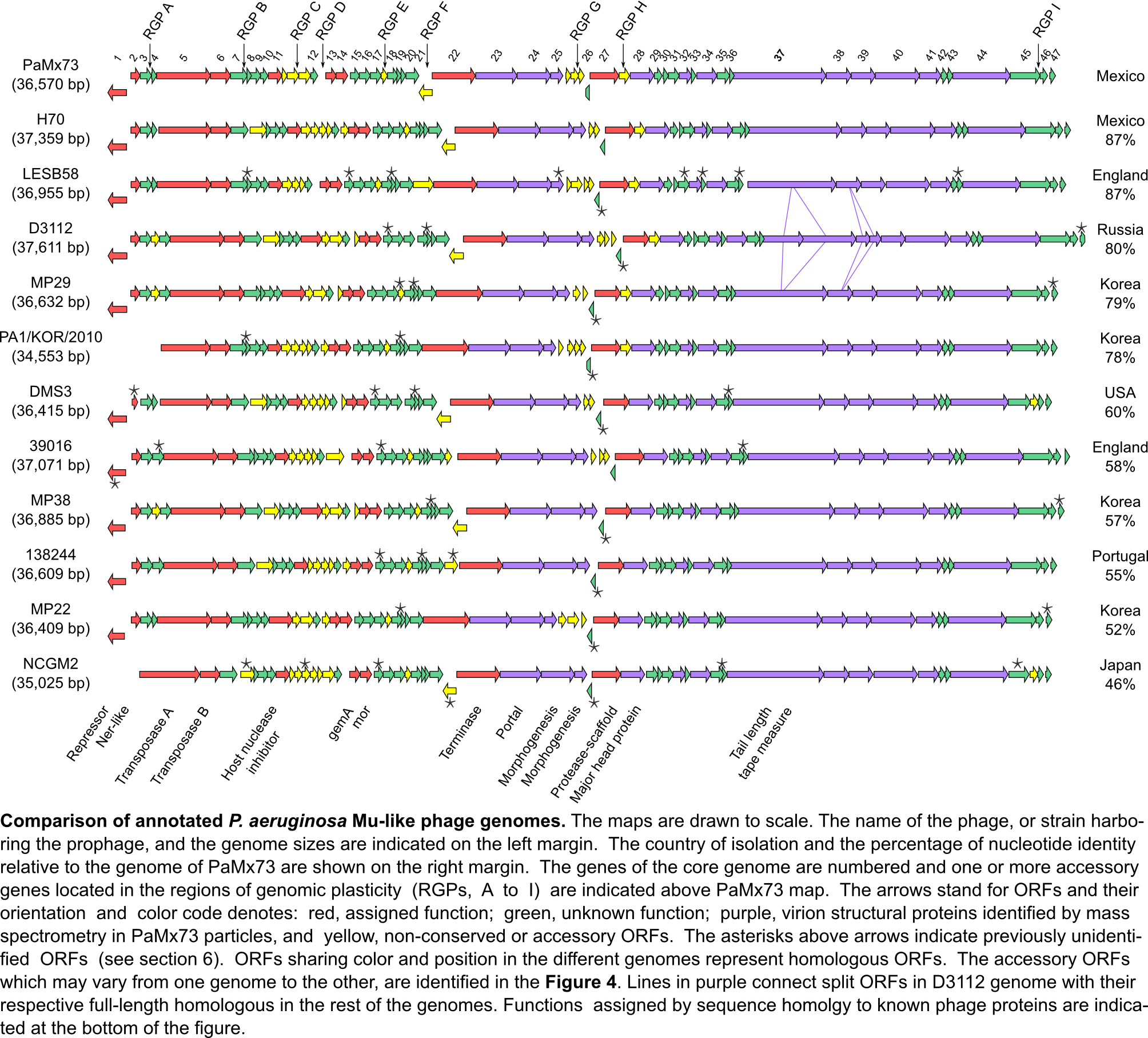

Supplement: Supplementary file 2 — Additional file 2: Comparison of annotated P. aeruginosa Mu-like phage genomes. (PNG 867 KB) [file 12864_2014_6884_MOESM2_ESM.png]
