# Supplementary material for: Core and accessory genome architecture in a group of Pseudomonas aeruginosa Mu-like phages
Source: BMC Genomics. 2014 Dec 19;15(1):1146. doi: 10.1186/1471-2164-15-1146 (PMC4378225; doi:10.1186/1471-2164-15-1146)
Supplement: Supplementary file 3 — Additional file 3: Transposase binding sites found in the phage genomes analyzed. (PDF 80 KB) [file 12864_2014_6884_MOESM3_ESM.pdf]

Additional file 3: Transposase binding sites found in the phage genomes analyzed

Phage	Putative trasposase binding sites					
	L1	L2	L3	R3	R2	R1*
PaMx73	GTGACTGTTTTGACACATGCCA (10)	GGGACAGTTTGGAAAAAATCGA (93)	AGCAAGGACTGCAAAATATTGA (124)	CGATGGCGATATCGTCGTGCTG (93)	CGCTGGCGAAACGGTTAGGGCG (46)	GCGCGGCGCGAAATTTGGCGCG (4)
H70	GTGACTGTTTTGACACATGCCA (10)	GCGGGTGTTTGGAAAATAACGA (93)	GGCGCGGAATGAAAGATATTGA (124)	CGATGGCGATATCGTCGTCCTG (93)	CGCTGGCGAAACGGTTAGGGCG (46)	GCGCGGCGCGAAATTTGGCGCG (4)
LESB58	GTGACTGTTTTGACACATGCCA (10)	GGGACAGTTTGGAAAAAATCGA (93)	AGCAAGGACTGCAAAATATTGA (124)	CGATGGCGATATCGTCGTGCTG (93)	CGCTGGCGAAACGGTTAGGGCG (46)	GCGCGGCGCGAAATTTGGCGCG (4)
D3112	GTGACTGTTTTGACACATGCCA (10)	GAGGGCGTTTGGAAAATATCGA (93)	AGCAAGGACTGCAAAATATTGA (124)	-	-	-
MP29	GTGACTGTTTTGACACACGCCA (10)	GCGGGTGTTTGGAAAATATCGA (93)	AGCAAGGACTGCAAAATATTGA (124)	CGATGGCGATATCGTCGTGCTG (93)	CGCTGGCGAAACGGTTAGGGCG (46)	GCGCGGCGCGAAATTTGGCGCG (4)
PA1/KOR	-	-	-	CGATGGCGATATCGTCGTGCTG (93)	CGCTGGCGAAACGGTTAGGGCG (46)	ACCGGGCGCGAAATTTGGCGCG (4)
DMS3	GTGACTGTTTTGACACATGCCA (10)	GGGACAGTTTGGAAAAAATCGA (93)	AGCAAGGAATGCAAAATATTGA (124)	CGACGGCGATATCGTCGTCCTG (93)	CGCTGGCGAAACGGTTAGGGCG (46)	GCGCGGCGCGAAATTTGGCGCC (4)
39016	GTGACTGTTTTGACACATGCCA (10)	GCGGGTGTTTGGAAAATATCGA (93)	AGCAAGGAATGATAGATATTGA (124)	CGATGGCGATATCGTCGTGCTG (93)	CGCTGGCGAAACGGTTAGGGCG (46)	GCGCGGCGCGAAATTTGGCGCG (4)
MP38	GTGACTGTTTTGACACATGCCA (10)	GCGGGTGTTTGGAAAATATCGA (93)	AGCAAGGAATGAAAGATATTGA (124)	CGATGGCGATATCGTCGTCCTG (93)	CGCTGGCGAAACGGTCAGGGCG (46)	GCGCGGCGCGAAATTTGGCGCG (4)
138244	GTGACTGTTTTGACACATGCCA (10)	GCGGGTGTTTGGAAAATATCGA (93)	AGCAAGGAATGATAGATATTGA (124)	CGATGGCGATATTGTCGTCCTG (93)	CGCAGCCGGAACGGCTAGGGCG (46)	GCGCGGCGCGAAATTTGGCGCG (4)
MP22	GTGACTGTTTTGACACATGCCA (10)	GCGGGTGTTTGGAAAATATCGA (93)	AGCAAGGAATGCAAAATATTGA (124)	CGATGGCGATATCGTCGTGCTG (93)	CGCTGGCGAAACGGTTAGGGCG (46)	GCGCGGCGCGAAATTTGGCGCG (4)
NCGM2	-	-	-	CGATGGCGATATCGTCGTGCTG (93)	CGCTGGCGAAACGGTTAGGGCG (46)	GCGCGGCGCGAAATTTGGCGCG (4)

In the putative transposase binding sites L stands for sites in the left and R for sites located in the right genome ends [4]. In parenthesis is indicated the number of bases from the corresponding genome end to the first nucleotide of the binding sites. The genome ends are considered as the first nucleotide in the left or right 5´TGTs (see the text, section 2). Nucleotides in red represent differences with the consensus in the corresponding position.

* R1 is the only repeat inverted relative to the other sites, i.e., the 5' end is read from the bottom DNA strand, not as the other five sites whose 5' ends were read from the top DNA strand.
