# Supplementary material for: Core and accessory genome architecture in a group of Pseudomonas aeruginosa Mu-like phages
Source: BMC Genomics. 2014 Dec 19;15(1):1146. doi: 10.1186/1471-2164-15-1146 (PMC4378225; doi:10.1186/1471-2164-15-1146)
Supplement: Supplementary file 4 — Additional file 4: Prediction of PaMx73 virion protein functions by computational modeling. (PDF 19 KB) [file 12864_2014_6884_MOESM4_ESM.pdf]

Additional file 4:

Prediction of PaMx73 virion protein functions by computational modeling

Virion Protein (Pangenome designation)	Model confidence score: C-score.	Alignment confidence scores:			Structural match:		
		TM-score	RMSD	Coverage	PDB accession number [chain]	Molecule	Organism
PaMx73_36 (accessory-ORF-h)	-3.07	0.539	2.1	0.629	1VD0 [A]	Head decoration protein	Bacteriophage lambda
PaMx73_41 (cORF-32)	0.01	0.635	2.53	0.774	3FZ2 [K]	Tail terminator protein	Bacteriophage lambda
PaMx73_43 (cORF-34)	-4.31	0.740	2.25	0.820	3LJY [A]	Putative adhesion	*Parabacteroides distasonis*
PaMx73_47 (cORF-38)	-2.52	0.834	1.97	0.896	3SUC [A]	Tail spike protein	Bacillus phage phi29
PaMx73_48 (cORF-39)	-2.63	0.830	2.04	0.889	3SUC [A]	Tail spike protein	Bacillus phage phi29
PaMx73_49 (cORF-40)	-1.47	0.927	1.37	0.945	3CM9 [S]	Immunoglobulin kappa light chain	*Homo sapiens*
PaMx73_50 (cORF-41)	-4.08	0.875	2.15	0.967	3PE7 [A]	Oligogalacturonate lyase	*Yersinia enterocolitica*
PaMx73_53 cORF-44	-0.95	0.889	1.29	0.903	4LGN [A]	Glycoside hydrolase	*Acidothermus cellulolyticus*

The 3D models of the virion proteins were obtained and compared with those deposited in the protein data bank using the web server of I-TASSER ([35], http://zhanglab.ccmb.med.umich.edu/I-TASSER/). Confidence scores of the model prediction (column 2) and comparison (columns 3-5) are explained in [35]. 3D models with poor quality scores, as defined by our cutoff values (see methods), are indicated in the gray-shaded cells.
