# Supplementary material for: Core and accessory genome architecture in a group of Pseudomonas aeruginosa Mu-like phages
Source: BMC Genomics. 2014 Dec 19;15(1):1146. doi: 10.1186/1471-2164-15-1146 (PMC4378225; doi:10.1186/1471-2164-15-1146)
Supplement: Supplementary file 5 — Additional file 5: Equivalence of core ORFs in the compared genomes. (PDF 90 KB) [file 12864_2014_6884_MOESM5_ESM.pdf]

Additional file 5: Equivalence of core ORFs in the compared genomes

Core genome *	PaMx73	H70	LESB58	D3112	MP29	PA1/KOR	DMS3	39016	MP38	138244	MP22	NCGM2
**1**	1	1	15491	p1	gp1	-	1		gp1	07058	1	-
**2**	2	2	15501	p2	gp2	-		000870026	gp2	07063	2	-
**3**	3	3	15511	p3	gp3	-	2	000870027	gp3	07068	3	-
**4**	4	4	15521	p5	gp5	-	3		gp5	07073	5	-
**5**	5	5	15531	p6	gp6	ADU15499	4	000870028	gp6	07078	6	5449
**6**	6	6	15541	p7	gp7	ADU15500	5	000870029	gp7	07083	7	5448
**7**	7	7	15551	p8	gp8	ADU15501	6	000870030	gp8	07088	8	5447
**8**	8	9		p10	gp9		8	000870031	gp10	07098	10	5446
**9**	9	10	15561	p11	gp10	ADU15502	9	000870032	gp11	07103	11	5445
**10**	10	11	15571	p12	gp11	ADU15503	10	000870033	gp12	07108	12	5444
**11**	11	12	15581	p13	gp12	ADU15504	11	000870034	gp13	07113	13	5443
**12**	15	17	15621	p16	gp15	ADU15509	16	000870039	gp16	07138	16	5437
**13**	16	19	15631	p18	gp17	ADU15511	18	000870041	gp18	07148	18	5436
**14**	17	20	15641	p19	gp18	ADU15512	19	000870042	gp19	07153	19	5435
**15**	18	21			gp19	ADU15513			gp20		19a	
**16**	19	22	15651	p20	gp20	ADU15514	20	000870043	gp21	07158	20	5434
**17**	20	23	15661	p21	gp21	ADU15515	21	000870044	gp22	07163	21	5433
**18**	22	25	15681	p22	gp22	ADU15517	22	000870046	gp24	07173	22	5431
**19**	23	26						000870047				5430
**20**	24	27	15691	p23	gp23	ADU15518	23	000870048	gp25	07178	23	5429
**21**	25	28	15701	p24	gp24	ADU15519	24	000870049	gp26	07183	24	5428
**22**	27	30	15721	p26	gp25	ADU15520	26	000870051	gp28	07188	26	5427
**23**	28	31	15731	p27	gp26	ADU15521	27	000870052	gp29	07193	27	5426
**24**	29	32	15741	p28	gp27	ADU15522	28	000870053	gp30	07198	28	5425

**25**	30	33		p29	gp28	ADU15523	29	000870054	gp31	07203	29	5424
**26**	34	36						000870058				
**27**	35	37	15791	p33	gp31	ADU15528	32	000870059	gp32	07208	33	5423
**28**	37	39	15811	p35	gp33	ADU15530	33	000870060	gp33	07213	35	5422
**29**	38	40	15821	p36	gp34	ADU15531	34	000870061	gp34	07218	36	5421
**30**	39	41	15831	p37	gp35	ADU15532	35	000870062	gp35	07223	37	5420
**31**	40	42		p38	gp36	ADU15533	36	000870063	gp36	07228	38	5419
**32**	41	43	15841	p39	gp37	ADU15534	37	000870064	gp37	07233	39	5418
**33**	42	44		p40	gp38	ADU15535	38	000870065	gp38	07238	40	5417
**34**	43	45	15851	p41	gp39	ADU15536	39	000870066	gp39	07243	41	5416
**35**	44	46	15861	p42	gp41	ADU15537	40	000870067	gp41	07248	42	5415
**36**	45	47		p43	gp40	ADU15538			gp40	07253	43	
**37**	46	48	15871	p44-p45	gp42	ADU15539	41	000870068	gp42	07258	45e	5414
**38**	47	49	15881	p46-p47	gp43	ADU15540	42	000870069	gp43	07263	46e	5413
**39**	48	50	15891	p48	gp44	ADU15541	43	000870070	gp44	07268	48	5412
**40**	49	51	15901	p49	gp45	ADU15542	44	000870071	gp45	07273	49	5411
**41**	50	52	15911	p50	gp46	ADU15543	45	000870072	gp46	07278	50	5410
**42**	51	53	15921	p51	gp47	ADU15544	46	000870073	gp47	07283	51	5409
**43**	52	54		p52	gp48	ADU15545	47	000870074	gp48	07288	52	5408
**44**	53	55	15931	p53	gp49	ADU15546	48	000870075	gp49	07293	53	5407
**45**	54	56	15941	p54	gp50	ADU15547	49	000870076	gp50	07298	54	
**46**	55	57	15951	p55	gp51	ADU15548	51	000870077	gp51	07303	55	5405
**47**	56	58	15961			ADU15549	52	000870078		07308		5404

* The core ORF numbers correspond to those in Figure 4. Numbers in each column are those assigned to the ORFs in the original GenBank files.
(-): indicates an absent ORF probably by deletion (see the text for details). A blank cell stands for a previously unidentified ORF (see Additional file 7 as reference).
