# Supplementary material for: Core and accessory genome architecture in a group of Pseudomonas aeruginosa Mu-like phages
Source: BMC Genomics. 2014 Dec 19;15(1):1146. doi: 10.1186/1471-2164-15-1146 (PMC4378225; doi:10.1186/1471-2164-15-1146)
Supplement: Supplementary file 6 — Additional file 6: BLASTp searches of the ORFs encoded in the pangenome of the P. aeruginosa Mu-like phages analyzed. (PDF 127 KB) [file 12864_2014_6884_MOESM6_ESM.pdf]

Additional file 6:
BLASTp searches of the ORFs encoded in the core genome of the *P. aeruginosa* Mu-like phage PaMx73

Core genome (Conserved ORFs)			
**Core ORF number**	**Putative product (Conserved domains found, acc. no., e-value)**	**Closest sequence in databases (Organism, e-value)**	**Taxonomic distribution of the homologues***
ORF 01	Repressor (HTH_3, PF01381, 1.4e-05; Peptidase_S24, PF00717, 4.5e-12; LexA/Signal peptidase, SSF51306, 1.3e-26)	CDM42423 (*Pseudomonas pseudoalcaligenes* CECT 5344, 1e-85)	A (30), B (687), C (1)
ORF 02	Ner-like protein (lambda repressor-like DNA-binding domains, SSF47413, 4.7e-19)	CDM42422 (*Pseudomonas pseudoalcaligenes* CECT 5344, 8e-28)	A (26), B (248)
ORF 03	Hypothetical protein	CDM42421 (*Pseudomonas pseudoalcaligenes* CECT 5344, 2e-76)	A (28), B (127)
ORF 04	Hypothetical protein	Orthologous ORFan	A (14), B (35)
ORF 05	Transposase A (rve, PF00665, 7.5e-16; HTH_Tnp_Mu_1, PF02316, 3.5e-30; Ribonuclease H-like, SSF53098, 4.3e-54; Mu-transpos_C, PF09299, 4.9e-21)	WP_021476436 (*Pseudogulbenkiania ferrooxidans*, 0.0)	A (36), B (683)
ORF 06	Transposase B (AAA, PF00004, 6.2e-05; P-loop containing nucleoside triphosphate hydrolases, SSF52540, 4.8e-15)	WP_003463714 (*Pseudomonas pseudoalcaligenes*, 1e-126)	A (35), B (428)
ORF 07	DNA-binding domain-containing protein (Winged HTH transcription repressor DNA-binding, G3DSA:1.10.10.10, 5.6e-06)	YP_004846877 (*Pseudogulbenkiania sp.* NH8B, 6e-45)	A (34), B (199)
ORF 08	Hypothetical protein	Orthologous ORFan	A (5)
ORF 09	Hypothetical protein	WP_010564674 (*Pseudomonas extremaustralis*, 3e-38)	A (38), B (222)
ORF 10	Hypothetical protein	WP_003463709 (*Pseudomonas pseudoalcaligenes*, 4e-23)	A (30), B (70)
ORF 11	Host nuclease inhibitor protein (Phage_Mu_Gam, PF07352, 5.7e-49)	WP_001405163 (*Escherichia coli*, 7e-76)	A (31), B (1068)
ORF 12	Hypothetical protein	Orthologous ORFan	A (29), B (61)
ORF 13	GemA (Mu Gp16, PF06252, 5.1e-30)	WP_003463693 (*Pseudomonas pseudoalcaligenes*, 4e-52)	A (32), B (682)
ORF 14	Mor transcription activator (Mor, PF08765, 6.7e-15; Homeodomain-like, SSF46689, 8.4e-18)	WP_003463692 (*Pseudomonas pseudoalcaligenes*, 1e-57)	A (32), B (670)
ORF 15	Hypothetical protein	WP_003463691 (*Pseudomonas pseudoalcaligenes*, 3e-38)	A (24), B (39)

Core genome (Conserved ORFs)			
**Core ORF number**	**Putative product (Conserved domains found, acc. no., e-value)**	**Closest sequence in databases (Organism, e-value)**	**Taxonomic distribution of the homologues***
ORF 16	Hypothetical protein	WP_003463690 (*Pseudomonas pseudoalcaligenes*, 3e-69)	A (30), B (124)
ORF 17	Hypothetical protein	WP_003463689 (*Pseudomonas pseudoalcaligenes*, 2e-71)	A (35), B (289)
ORF 18	Hypothetical protein	WP_024360729 (*Klebsiella oxytoca*, 3e-63)	A (30), B (71)
ORF 19	Hypothetical protein	KCB35295 (*Bordetella hinzii* 8-296-03, 1e-06)	A (13), B (5)
ORF 20	Hypothetical protein	WP_003463686 (*Pseudomonas pseudoalcaligenes*, 6e-16)	A (30), B (54)
ORF 21	Hypothetical protein	YP_001119154 (*Burkholderia vietnamiensis* G4, 2e-73)	A (39), B (650)
ORF 22	Terminase large subunit (Archaeophage PsiM2 terminase large subunit, TIGR01630, 4.4e-30)	WP_003463679 (*Pseudomonas pseudoalcaligenes*, 0.0)	A (39), B (702)
ORF 23	Portal protein (Phage_Mu_Gp29, IPR009279, 6e-194)	WP_003463674 (*Pseudomonas pseudoalcaligenes*, 0.0)	A (38), B (751)
ORF 24	Virion morphogenesis protein (Phage_Mu_F, PF04233, 9.4e-33; phageSPP1_gp7, TIGR01641, 7.9e-21)	WP_003463671 (*Pseudomonas pseudoalcaligenes*, 0.0)	A (35), B (579)
ORF 25	Virion morphogenesis protein (Phage_tail_S, PF05069, 6.1e-32; Tail_comp_S, TIGR01635, 4.2e-15)	WP_003463668 (*Pseudomonas pseudoalcaligenes*, 6e-47)	A (42), B (756)
ORF 26	Hypothetical protein	Orthologous ORFan	A (1)
ORF 27	Protease (I) and scaffold (Z) proteins (Predicted protease, PIRSF016624, 3.6e-68; Mu-like_Pro, PF10123, 2.9e-95)	WP_003463665 (*Pseudomonas pseudoalcaligenes*, 3e-160)	A (34), B (683), C (1)
ORF 28	Major head subunit protein (Mu-like_gpT, PF10124, 7.9e-55)	WP_024302228 (*Pseudogulbenkiania* sp. MAI-1, 5e-141)	A (17), B (498)
ORF 29	Hypothetical protein	WP_024762343 (*Pseudomonas nitroreducens*, 3e-10)	A (13), B (26)
ORF 30	Hypothetical protein	WP_020306283 (*Pseudomonas stutzeri*, 9e-6)	A (29), B (49)
ORF 31	Hypothetical protein	WP_003463660 (*Pseudomonas pseudoalcaligenes*, 2e-67)	A (34), B (823)
ORF 32	Tail terminator protein	WP_003463658 (*Pseudomonas pseudoalcaligenes*, 7e-75)	A (30), B (146), C (1)

Core genome (Conserved ORFs)			
**Core ORF number**	**Putative product (Conserved domains found, acc. no., e-value)**	**Closest sequence in databases (Organism, e-value)**	**Taxonomic distribution of the homologues***
ORF 33	Hypothetical protein	CDM42387 (*Pseudomonas pseudoalcaligenes* CECT 5344, 3e-19)	A (30), B (52)
ORF 34	Major tail protein	WP_003463656 (*Pseudomonas pseudoalcaligenes*, 1e-90)	A (38), B (482)
ORF 35	Hypothetical protein	WP_003463653 (*Pseudomonas pseudoalcaligenes*, 2e-55)	A (41), B (235)
ORF 36	Hypothetical protein	Orthologous ORFan	A (10), B (12)
ORF 37	Tail length tape measure protein (Tape_meas_lam_C, PF09718, 1.1e-28, tape_meas_lam_C, TIGR01541, 4.7e-31, TMP_2, PF06791, 1.7e-38)	YP_235857 (*Pseudomonas syringae* pv. syringae B728a, 0.0)	A (18), B (416)
ORF 38	Virion structural protein	YP_164087 (*Pseudomonas* phage B3, 0.0)	A (40), B (269)
ORF 39	Virion structural protein	YP_164088 (*Pseudomonas* phage B3, 0.0)	A (40), B (220)
ORF 40	Virion structural protein	YP_164089 (*Pseudomonas* phage B3, 0.0)	A (54), B (302)
ORF 41	Virion structural protein (Phg, TIGR02218, 2.9e-90, Phage_BR0599, PF09356, 1.8e-19)	WP_024762350 (*Pseudomonas nitroreducens*, 1e-124)	A (80), B (325)
ORF 42	Hypothetical protein	WP_019361503 (*Pseudomonas fuscovaginae*, 4e-36)	A (57), B (234)
ORF 43	Hypothetical protein	WP_024762352 (*Pseudomonas nitroreducens*, 2e-31)	A (34), B (182)
ORF 44	Virion structural protein	WP_019361505 (*Pseudomonas fuscovaginae*, 0.0)	A (61), B (387)
ORF 45	Hypothetical protein	YP_164094 (*Pseudomonas* phage B3, 4e-62)	A (29), B (166)
ORF 46	Hypothetical protein	YP_008932592 (*Pseudomonas sp.* TKP, 6e-13)	A (30), B (194)
ORF 47	Hypothetical protein	YP_164095 (*Pseudomonas* phage B3, 6e-41)	A (22), B (158)

The core ORFs were present in all the genomes but those of PaMx73 were used as query in the BLASTp searches. Core ORF numbers are as in Figure 4. In the third column are indicated the best matches to the query except those of *P. aeruginosa* Mu-like phages. Virion structural proteins identified by mass spectrometry in PaMx73 are indicated in the gray-shaded lines.

*The taxonomic distribution of the total number of homologues found in the (nr) database of NCBI for the corresponding ORF is indicated. Categories A, B, and C denote the number of homologues (in parenthesis) found in Viruses, Bacteria and Eukaryota, respectively (see Methods). Note that the matches with Eukayota correspond to unpublished records.

BLAST-P searches of the accessory ORFs encoded in the *P. aeruginosa* Mu-like phages analyzed in this study

Accessory genome (Non-conserved ORFs)				
**Variable region (accessory ORF) ***	**Gene (phage) ****	**Putative product (Conserved domains found, acc. no., e-value) [size in amino acids]**	**Closest sequence in databases (Organism, e-value)**	**Taxonomic distribution of the homologues*****
RGP 1 (a)	ORF 4 (D3112)	Hypothetical protein [108]	Orthologous ORFan	A (13), B (4)
RGP 2 (b)	ORF 9 (D3112)	Hypothetical protein [211]	AFQ22082 (*Pseudomonas* phage YMC/01/01/P52_PAE_BP, 2e-20)	A (17), B (66)
RGP 3 (c1)	ORF 12 (PaMx73)	Hypothetical protein [67]	NP_901832 (*Chromobacterium violaceum* ATCC 12472, 3e-04)	A (5), B (18)
RGP 3 (c2)	ORF 13 (PaMx73)	Hypothetical protein [148]	WP_005789428 (*Pseudomonas synxantha*, 2e-43)	A (1), B (6)
RGP 3 (c3)	ORF 12 (DMS3)	Hypothetical protein [93]	WP_010564671 (*Pseudomonas extremaustralis*, 6e-18)	A (10), B (293)
RGP 3 (c4)	15591 (LESB58)	Hypothetical protein [125]	CDF82111 (*Pseudomonas knackmussii* B13, 6e-47)	A (10), B (59)
RGP 3 (c5)	ORF 14 (D3112)	Hypothetical protein [102]	WP_006162149 (*Cupriavidus basilensis,* 6e-07)	A (26), B (34)
RGP 3 (c6)	15601 (LESB58)	Hypothetical protein [93]	WP_024767768 (*Pseudomonas nitroreducens*, 2e-27)	A (17), B (53)
RGP 3 (c7)	15611 (LESB58)	Hypothetical protein [68]	WP_009405478 (*Pseudomonas putida*, 2e-20)	A (18), B (97)
RGP 3 (c8)	ORF 14 (PaMx73)	Hypothetical protein [149]	WP_010564670 (*Pseudomonas extremaustralis*, 2e-30)	A (6), B (104)
RGP 4 (d1)	ORF 17 (D3112)	Hypothetical protein [60]	WP_003463697 (*Pseudomonas pseudoalcaligenes*, 1e-29)	A (12)
RGP 4 (d2)	000870040 (39016)	Hypothetical protein [223]	WP_024767764 (*Pseudomonas nitroreducens*, 7e-102)	A (7), B (781)
RGP 5 (e)	ORF 21 (PaMx73)	Hypothetical protein [80]	Orthologous ORFan	A (18), B (54)
RGP 6 (f1)	ORF 26 (PaMx73)	Hypothetical protein [168]	WP_017629234 (*Neisseria meningitidis*, 1e-24)	A (20), B (245)
RGP 6 (f2)	15711 (LESB58)	PAPS-reductase domain-containing protein (PAPS-reductase, cd01713, 1.70e-04) [258]	WP_024668829 (*Pseudomonas syringae*, 2e-146)	A (13), B (193)
RGP 6 (f3)	000870050 (39016)	Hypothetical protein [83]	Orthologous ORFan	A (3), B (8)

Accessory genome (Non-conserved ORFs)				
**Variable region (accessory ORF) ***	**Gene (phage) ****	**Putative product (Conserved domains found, acc. no., e-value) [size in amino acids]**	**Closest sequence in databases (Organism, e-value)**	**Taxonomic distribution of the homologues*****
RGP 6 (f4)	13046-13639 (138244)	Hypothetical protein [197]	WP_010378731 (*Xanthomonas campestris*, 2e-77)	B (44)
RGP 7 (g1)	ORF 31 (PaMx73)	Type I-E anti-CRISPR protein [65]	YP_950454 (*Pseudomonas* phage DMS3, 4e-37) Orthologous ORFan	A (6), B (13)
RGP 7 (g2)	ORF 34 (H70)	Hypothetical protein [67]	YP_009007134 (Pseudomonas phage vB_PaeP_Tr60_Ab31, 8e-41)	A (3), B (4)
RGP 7 (g3)	ORF 30 (D3112)	Type I-F anti-CRISPR protein [90]	YP_002332454 (*Pseudomonas* phage MP29, 2e-43) Orthologous ORFan	A (6), B (5)
RGP 7 (g4)	ORF 29a (MP22)	Type I-E anti-CRISPR protein [98]	YP_007392439 (*Pseudomonas* phage JBD88a, 3e-55) Orthologous ORFan	A (5), B (5)
RGP 7 (g5)	ORF 32 (PaMx73)	Type I-F anti-CRISPR protein [100]	AEY99442 (*Pseudomonas* phage JBD26, 7e-68) Orthologous ORFan	A (4), B (8)
RGP 7 (g6)	15751 (LESB58)	Type I-E anti-CRISPR protein [54]	NP_938238 (*Pseudomonas* phage D3112, 3e-25) Orthologous ORFan	A (6), B (12)
RGP 7 (g7)	15761 (LESB58)	Type I-F anti-CRISPR protein [139]	YP_007392739 (*Pseudomonas* phage JBD5, 7e-92) Orthologous ORFan	A (9), B (49)
RGP 7 (g8)	15771 (LESB58)	Type I-F anti-CRISPR protein [51]	YP_007392740 (*Pseudomonas* phage JBD5, 9e-17) Orthologous ORFan	B (13)
RGP 7 (g9)	ORF 33 (PaMx73)	DNA-binding domain-containing protein (*Lambda repressor-like, DNA-binding domain, IPR010982, 3.8e-7*) [74]	WP_004904558 (*Acinetobacter brisouii*, 3e-21)	A (20), B (119)
RGP 8 (h)	ORF 36 (PaMx73)	Head decoration protein [132]	WP_014258878 (*Desulfovibrio africanus*, 2e-47)	A (18), B (490)
RGP 9 (i)	ORF 50 (DMS3)	Hypothetical protein [100]	YP_009030615 (*Pseudomonas* phage KPP25, 4e-46)	A (10), B (464)

* The variable regions and accessory ORFs are as in Figure 4.
** ORF number as in the original GenBank file used as query in the BLASTp searches.
*** The taxonomic distribution of the total number of homologues found in the (nr) database of NCBI for the corresponding ORF is indicated. Categories A and B denote the number of homologues (in parenthesis) found in Viruses and Bacteria, respectively (see Methods).
Note that the structural protein identified by mass spectrometry in the virion of PaMx73 is indicated in the gray-shaded line.
