# Supplementary material for: Core and accessory genome architecture in a group of Pseudomonas aeruginosa Mu-like phages
Source: BMC Genomics. 2014 Dec 19;15(1):1146. doi: 10.1186/1471-2164-15-1146 (PMC4378225; doi:10.1186/1471-2164-15-1146)
Supplement: Supplementary file 7 — Additional file 7: Overlooked ORFs in the GenBank files of P. aeruginosa Mu-like phages. (PDF 95 KB) [file 12864_2014_6884_MOESM7_ESM.pdf]

Additional file 7: Overlooked ORFs in the GenBank files of *P. aeruginosa* Mu-like phages

Phage	ORF identified [a]	Location [b]	Strand [c]	Length [d] (amino acids)	Predicted function of the gene product [e]
**LESB58**	8	5513-5629	+	38	
	15	9244-9591	+	115	
	19	11055-11168	+	37	
	25	17181-17648	+	155	Virion morphogenesis protein
	26	19007-18891	-	38	
	31	22027-22440	+	138	
	33	22907-23086	+	59	
	36	24378-24506	+	42	
	43	32716-32934	+	72	
**D3112**	15	10749-11096	+	115	
	19	12436-12549	+	37	
	26	19786-19670	-	38	
	47	37413-37610	+	66	Hypothetical protein (Incomplete protein probably due to missing sequence)
**MP29**	e	11294-11536	+	80	
	19	11927-12040	+	37	
	26	18694-18578	-	38	
	47	36321-36545	+	74	
**PA1/KOR**	8	3402-3518	+	38	
	19	9314-9427	+	37	
	26	16611-16531	-	26	Hypothetical protein (Incomplete protein by premature stop codon)
**DMS3**	2	1065-1280	+	71	Ner-like protein (Shorter protein probably by assembly error)
	15	10261-10608	+	115	
	19	11949-12062	+	37	
	26	19029-18913	-	38	
	36	23999-24127	+	42	
**39016**	1	847-170	-	225	Repressor
	4	1881-2312	+	143	

	15	10463-10810	+	115	
	36	24571-24699	+	42	
**MP38**	19	12569-12682	+	37	
	26	19106-18990	-	38	
	47	36574-36798	+	74	
**138244**	15	10420-10767	+	115	
	19	12231-12344	+	37	
	f4	13046-13639	+	197	
	26	18825-18709	-	38	
**MP22**	19	11438-11551	+	37	
	26	18644-18528	-	38	
	47	36098-36319	+	74	
**NCGM2**	b	3808-4383	+	191	
	c5	6230-6538	+	102	
	15	8993-9340	+	115	
	f1	12152-11628	-	175	
	26	17329-17213	-	38	
	36	22380-22508	+	42	
	45	33273-34118	+	281	

[a] The numbers or low case letter codes were assigned in this work for the core or accessory genomes, respectively (see pangenome, Figure 4).

[b] Locus coordinates in the reported genome.

[c] + and – indicate right and left transcription direction, respectively, as the genome maps are represented here.

[d] Number of amino acids in the predicted protein.

[e] The blank cells correspond to hypothetical proteins of unknown function.
